# The northern limit of corals of the genus *Acropora* in temperate zones is determined by their resilience to cold bleaching

**DOI:** 10.1038/srep18467

**Published:** 2015-12-18

**Authors:** Tomihiko Higuchi, Sylvain Agostini, Beatriz Estela Casareto, Yoshimi Suzuki, Ikuko Yuyama

**Affiliations:** 1 Graduate School of Science and Technology, Shizuoka University, Shizuoka, Japan; 2 Shimoda Marine Research Center, University of Tsukuba, Shizuoka, Japan; 3 Research Institute of Green Science and Technology, Shizuoka University, Shizuoka, Japan; 4 Center for Information Biology, National Institute of Genetics, Shizuoka, Japan

## Abstract

The distribution of corals in Japan covers a wide range of latitudes, encompassing tropical to temperate zones. However, coral communities in temperate zones contain only a small subset of species. Among the parameters that determine the distribution of corals, temperature plays an important role. We tested the resilience to cold stress of three coral species belonging to the genus *Acropora* in incubation experiments. *Acropora pruinosa*, which is the northernmost of the three species, bleached at 13 °C, but recovered once temperatures were increased. The two other species, *A*. *hyacinthus* and *A*. *solitaryensis*, which has a more southerly range than *A*. *pruinosa*, died rapidly after bleaching at 13 °C. The physiological effects of cold bleaching on the corals included decreased rates of photosynthesis, respiration, and calcification, similar to the physiological effects observed with bleaching due to high temperature stress. Contrasting hot bleaching, no increases in antioxidant enzyme activities were observed, suggesting that reactive oxygen species play a less important role in bleaching under cold stress. These results confirmed the importance of resilience to cold stress in determining the distribution and northern limits of coral species, as cold events causing coral bleaching and high mortality occur regularly in temperate zones.

Hermatypic corals typically thrive year-round in tropical areas with warm, clear waters. However, high-latitude reefs and coral communities occur in some areas, including the southern coast of Japan mainland^1^. The distribution of corals along the Pacific coast of Japan ranges from the southernmost islands of the Ryukyu Archipelago (24 °N) to Amatsukominato in Chiba Prefecture (34 °N, Supplementary Fig. S1)^2^,^3^. The presence of hermatypic corals at these high latitudes is due mainly to the strong Kuroshio current, which brings warm water as far as Tateyama^1^. Factors limiting the growth of hermatypic corals include temperature, the aragonite saturation state of calcium carbonate, salinity, nutrient concentrations, light penetration, currents, and competition with other biota^2^,^4^. In particular, seawater temperature strongly affects the survival of reef-building corals and their symbionts, and ecological surveys suggest that it is a major determinant of these organisms’ latitudinal distributions^4^. Acroporid corals are abundant in the tropics and exhibit the highest diversity among corals, with approximately 180 species in the genus *Acropora*^5^. In Japan, acroporid species can be observed even in the northernmost reefs, although their diversity decreases significantly at the highest latitudes. The low-temperature limit for temperate species dominating high-latitude coral communities in Japan has been estimated at 13 °C^6^. At the latitude extreme near Tateyama (34 °N), *Acropora pruinosa* largely dominates the coral community, while *A*. *solitaryensis* is present in lower numbers. The abundance of *A*. *solitaryensis* increases in more southerly waters, while *A*. *hyacinthus* thrives south of Kushimoto (33 °N)^1^,^7^. Temperature at the highest-latitude *Acropora* habitats in Japan ranged from 13.3–25.6 °C (34 °N, Tateyama) in 2013. At Shirahama (33 °N), the temperature ranged from 14.4–28.0 °C^8^, and in sub-tropical areas where *A*. *hyacinthus* is common, it ranged from 20.2–29.3 °C (26°N, Sesoko) (Supplementary Table S1).

Cold temperature stress can cause bleaching in corals, and sometimes mass mortality^9^. With prolonged exposure, experiments have shown that cold temperatures lead to higher mortality rates in tropical corals than high temperatures^10^. Cold bleaching is often seen in both the tropical^11^ and temperate zones^12^. Bleaching of *A*. *solitaryensis* and other tabular acroporids was reported from high-latitude coral communities near Nagasaki (32.5 °N) after 12 days of low temperatures (<13 °C) in 2013^12^. Compared to bleaching caused by high temperature stress, information on the mechanisms of cold stress bleaching is limited. Studies have demonstrated that cold stress bleaching can impair the photosynthetic apparatus of zooxanthellae, as cold-bleached corals have low photosynthetic quantum yields (*F*_*v*_*/F*_*m*_)^11^,^13^, low photosynthetic rates^14^, low chlorophyll contents^13^, and reduced zooxanthellae densities^15^. The physiological effects of cold stress on coral hosts are less well-documented, although studies have reported reduced growth and feeding rates at low temperatures^15^,^16^.

Experiments investigating the mechanisms of cold bleaching have varied in their selection of minimum temperatures, tropical or temperate study species, and the duration of exposure to cold stress. Therefore, a more complete physiological examination of the effects of cold bleaching on temperate corals is needed to understand the role it plays in determining their latitudinal distributions. This study examined the resilience to cold stress of three *Acropora* coral species established at different latitudes in Japan. We investigated the physiological effects of cold stress on these corals, and the mechanisms of any resulting cold bleaching, by means of laboratory experiments.

## Results

### Bleaching state

**Figure 1:**
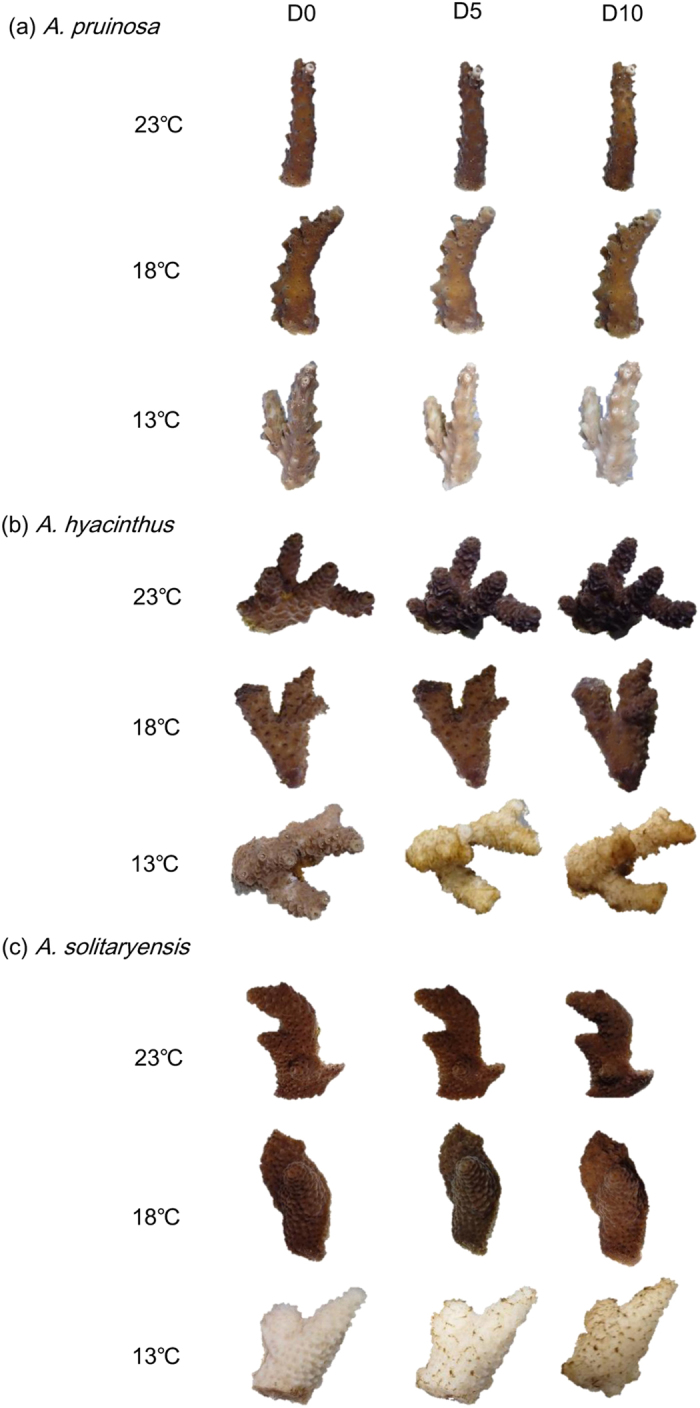
Photographs of acroporid corals after incubation at each temperature. (**a**) *Acropora pruinosa* at 23, 18, and 13 °C; (**b**) *A. hyacinthus* at 23, 18, and 13 °C; (**c**) *A. solitaryensis* at 23, 18, and 13 °C.

**Figure 2:**
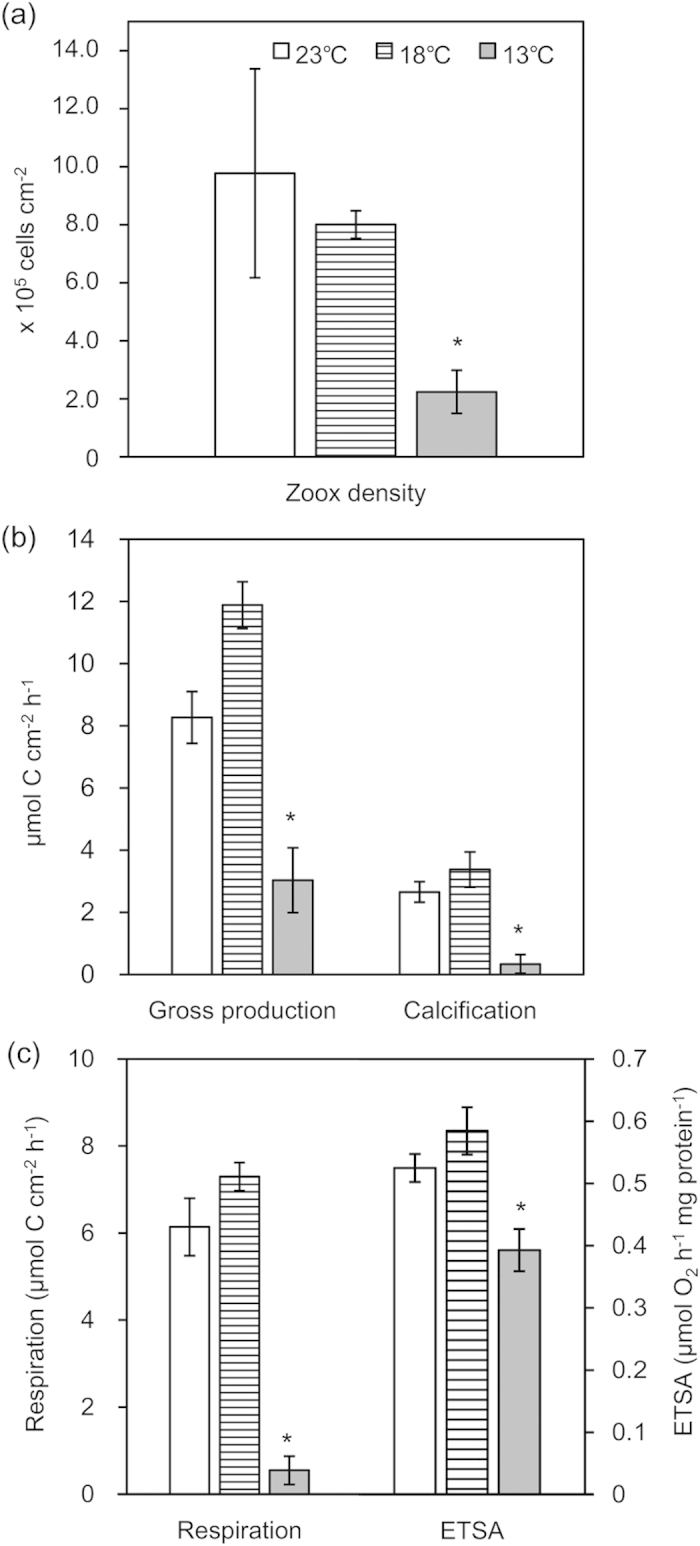
(**a**) Zooxanthellae density, (**b**) gross production and calcification, (**c**) respiration and respiratory electron transport system activity (ETSA) of *Acropora pruinosa* after 10 days at 23, 18, and 13 °C. Asterisks (*) indicate P < 0.05 compared to the control (18 °C) based on a Tukey-Kramer HSD test.

All corals survived for 10 days at higher temperatures of 18 °C and 23 °C (Fig. 1). No visible bleaching was observed in the fragments of all three species at 18 °C or 23 °C, while severe bleaching (*A*. *pruinosa*) and mortality (*A*. *solitaryensis* and *A*. *hyacinthus*) occurred at 13 °C. *A. pruinosa* gradually bleached throughout the incubation period at 13 °C (Fig. 1a). On day 0, *A*. *hyacinthus* bleaching was not severe (Fig. 1b), whereas *A*. *solitaryensis* was already severely bleached by day 0 (i.e., bleaching occurred during the gradual temperature decrease from 18 °C to 13 °C). Both *A*. *hyacinthus* and *A*. *solitaryensis* were dead within 5 days at 13 °C, after which macroalgae began to attach to their coral skeletons (Fig. 1b,c). Only *A*. *pruinosa* survived after 10 days at 13 °C. The zooxanthellae densities of the three species did not significantly change after 10 days at 18 °C or 23 °C (Table 1). By contrast, the zooxanthellae density of *A*. *pruinosa* decreased significantly by 77% at 13 °C compared to the control of 18 °C (HSD, P < 0.05; Fig. 2a).

### Physiological response of corals

**Figure 3:**
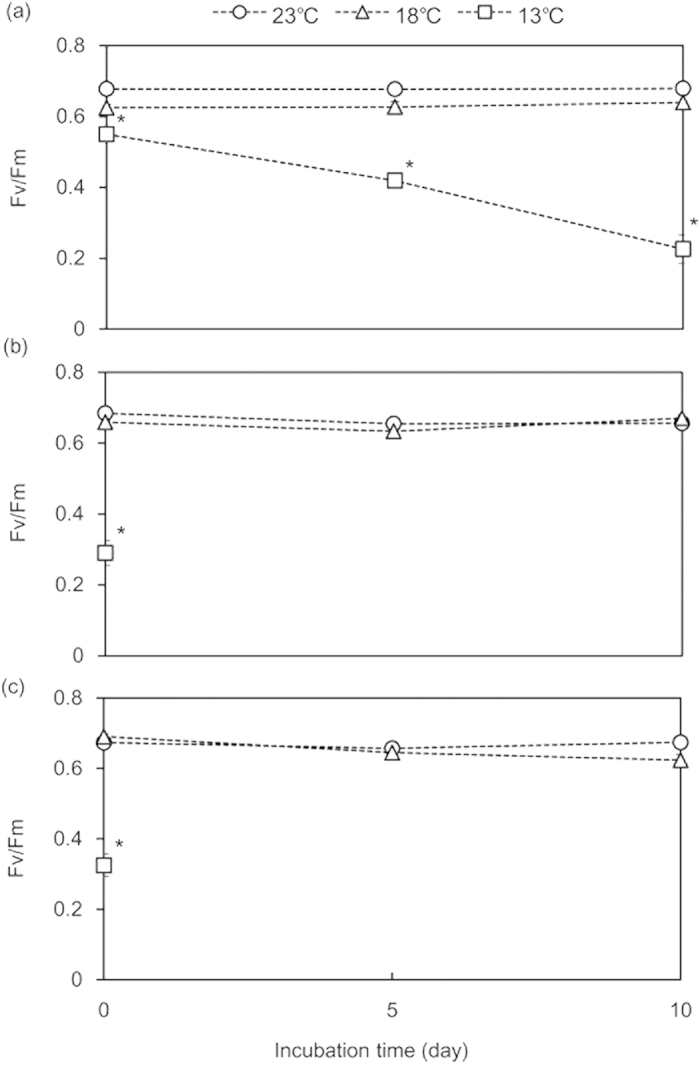
Variation in the photosynthetic maximum quantum yield (*F*_*v*_*/F*_*m*_) of acroporid corals. (**a**) *Acropora pruinosa* at 23, 18, and 13 °C; (**b**) *A. hyacinthus* at 23, 18, and 13 °C; (**c**) *A. solitaryensis* at 23, 18, and 13 °C. Asterisks (*) indicate P < 0.05 compared to the control (18 °C) based on a Tukey-Kramer HSD test.

The *F*_*v*_*/F*_*m*_ ratios of all three *Acropora* species remained stable at 0.6–0.7 at 18 °C and 23 °C over 10 days, but decreased significantly at 13 °C (Fig. 3). The *F*_*v*_*/F*_*m*_ ratios of *A*. *pruinosa* significantly decreased at 13 °C, with average ( ± SE) values of 0.55 ± 0.02, 0.42 ± 0.01, 0.23 ± 0.04 on day 0, 5, and 10, respectively (HSD, P < 0.05). The *F*_*v*_*/F*_*m*_ ratios of *A*. *hyacinthus* and *A*. *solitaryensis* had decreased to 0.29 ± 0.03 and 0.33 ± 0.03, respectively, by day 0 (HSD, P < 0.05).

The rates of gross production, respiration, and calcification of all three species did not significantly differ at 18 °C and 23 °C (Table 1). However, all three rates of *A*. *pruinosa* significantly decreased at 13 °C (Fig. 2b,c). In particular, the respiration rate (7.3 ± 0.9 μmol cm^−2^ h^−1^ at 18 °C vs. 0.5 ± 0.3 μmol cm^−2^ h^−1^ at 13 °C) and calcification rate (3.4 ± 0.6 μmol cm^−2^ h^−1^ at 18 °C vs. 0.3 ± 0.3 μmol cm^−2^ h^−1^ at 13 °C) were close to zero at 13 °C. The mitochondrial electron transport activity (ETSA) of *A*. *pruinosa* also significantly decreased at 13 °C (HSD, P < 0.05; Fig. 2c). The ETSA of all three *Acropora* species did not significantly differ between 18 °C and 23 °C (Table 1).

### Antioxidant enzyme activities

**Figure 4:**
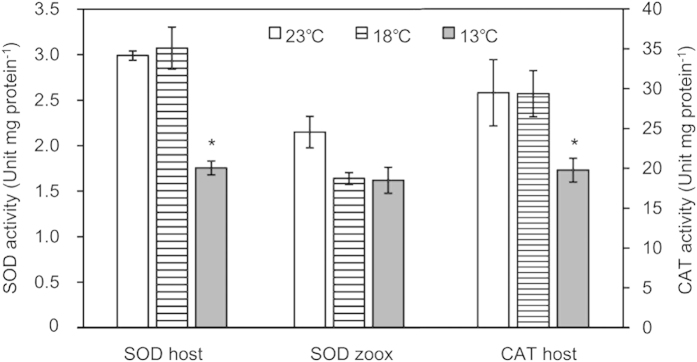
Antioxidant enzyme activities (superoxide dismutase: SOD, catalase: CAT) in host coral tissue and zooxanthellae (zoox) of *Acropora pruinosa* after 10 days at 23, 18, and 13 °C. Asterisks (*) indicate P < 0.05 compared to the control (18 °C) based on a Tukey-Kramer HSD test.

The activities of the antioxidant enzymes superoxide dismutase (SOD) and catalase (CAT) are shown in Table 1. The SOD and CAT activities of host coral and zooxanthellae did not significantly differ between 18 °C and 23 °C for *A*. *pruinosa*. In contrast, the SOD and CAT activities of *A*. *pruinosa* host coral significantly decreased in the 13 °C treatment (HSD, P < 0.05; Fig. 4). Moreover, host and zooxanthellae SOD activity of *A*. *solitaryensis* and zooxanthellae SOD activity of *A*. *hyacinthus* were significantly lower at 18 °C compared to 23 °C (HSD, P < 0.05; Table 1). Host CAT activity of *A*. *solitaryensis* and *A*. *hyacinthus* did not differ between 18 °C and 23 °C.

### Recovery from cold stress

**Figure 5:**
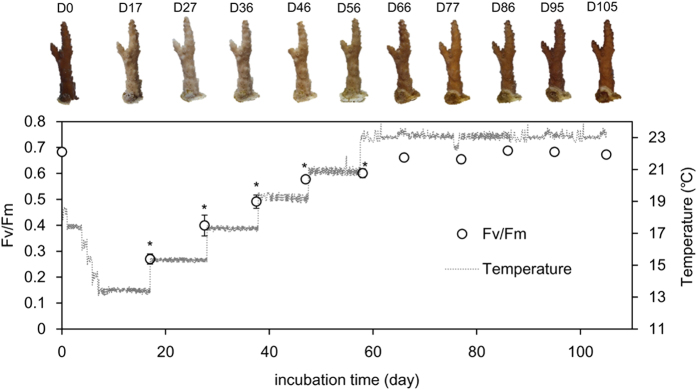
Variation in the photosynthetic maximum quantum yield (*F*_*v*_*/F*_*m*_), temperature and photograph of *Acropora pruinosa*. Asterisks (*) indicate P < 0.05 compared to the initial value of *F*_*v*_*/F*_*m*_ at 18 °C based on a Dunnett’s test. The dotted line indicates water temperature.

*A. pruinosa* experienced bleaching under low temperature stress (Fig. 5). Photosynthetic quantum yield (*F*_*v*_*/F*_*m*_) was significantly lower on day 17 (13 °C), day 27 (15 °C), day 36 (17 °C), day 46 (19 °C), and day 56 (21 °C), compared to initial values on day 0 (18 °C) (Dunnett’s test, P < 0.05). *F*_*v*_*/F*_*m*_ gradually increased with increased temperature, and values did not significantly differ after day 66. The color of *A*. *pruinosa* became pale at 13 °C and gradually turned brown with increased temperature. All three replicates of bleached *A*. *pruinosa* survived for 100 days when temperatures were increased, and corals completely recovered throughout the incubation.

## Discussion

*A. pruinosa* is endemic to high-latitude zones^7^, and dominates the acroporid corals at these latitudes on both the Pacific and Sea of Japan coasts^17^. Our results clearly indicate that *A*. *pruinosa* has high tolerance to cold temperatures. Suzuki *et al.*^12^ reported that *A*. *solitaryensis* and *A*. *hyacinthus* bleached and died within 12 days of cold stress below 13 °C in the field, yet *A*. *pruinosa* survived after this period. Average temperatures in Izu and Tateyama, Japan, where *A*. *pruinosa* dominates, are 13–14 °C over the winter months, with minimum temperatures as low as 10 °C^3^. Even though the stress response variation among different genotypes within each species may have been underestimated due to the use of a single genotype per species, our results suggest that among the three species tested, only *A*. *pruinosa* can survive at these temperatures, allowing this species to dominate at these locations. To understand the lower abundance and even absence of *A*. *pruinosa* at lower latitudes, the effects of high temperature on its physiology must be understood.

In our experiment, *A*. *hyacinthus* and *A*. *solitaryensis* exhibited minimal differences in cold stress resilience. At the lowest temperature of 13 °C, both species died, clearly demonstrating that they are less resistant to cold stress than *A*. *pruinosa*. These results are consistent with field observations in which *A*. *pruinosa* survived water temperatures <13 °C^12^. This low resistance to cold stress is sufficient to explain why *A*. *hyacinthus* and *A*. *solitaryensis* are less common at higher latitudes than *A*. *pruinosa*, although our results do not elucidate fine-scale variation in their distributions. The geographic distribution of these two species overlap^7^,^17^, but their relative abundance varies latitudinally, with *A*. *hyacinthus* dominating the lower latitudes. A finer-scale temperature range and different exposure times to cold stress should be tested to reveal any differences in cold-stress resilience between *A*. *hyacinthus* and *A*. *solitaryensis*. However, their physiological responses to cold stress may not differ substantially and other factors may play a larger role in determining the fine-scale distribution of these species.

The most widely accepted mechanism of hot bleaching is the onset of the photoinhibition of photosynthesis in zooxanthellae^18^. Photoinhibition is caused by a reduction in photosynthetic electron transport combined with a continued high absorption of excitation energy, resulting in increased production of reactive oxygen species (ROS). Both hot and cold bleaching are induced by light under stressful temperatures. Previous experiments have demonstrated that at cold temperatures, zooxanthella photosynthetic efficiency (*F*_*v*_*/F*_*m*_) decreases only under well-lit conditions^13^. Under cold stress in the light, ROS such as singlet oxygen and superoxide anion radicals are generated by the photosynthetic electron transport chain in plant chloroplasts^19^ due to an imbalance between the amount of light energy absorbed and processed by enzymes in PSII^15^. Increased ROS production is typically accompanied by an increase in SOD and CAT activities under high temperature stress^20^. In our study, SOD and CAT activities in host coral decreased at low temperatures, and we detected no significant increase in zooxanthella SOD. These results can be interpreted in two ways: 1) corals and zooxanthellae do not have the capacity to increase their antioxidant enzyme activities under cold stress, resulting in severe damage and high mortality; or 2) ROS levels do not increase at low temperatures. While the SOD activity supports both hypotheses, the decrease in host CAT activity may be the result of decreased *in vivo* production of hydrogen peroxide by SOD due to decreased SOD activity and the temperature-dependency of the reaction^21^,^22^. In rice cultivars, high antioxidant enzyme activity is associated with high cold-stress tolerance^23^. The lack of increase in antioxidant enzyme activity in both coral and zooxanthellae under cold stress suggests that corals do not have strong tolerance to low temperatures, which is supported by the observed high mortality rate. Under high temperatures, bleaching is the final defense of corals against oxidative stress and functions to reduce levels of ROS that are produced by zooxanthellae^24^. If cold stress results in the production of ROS and corals and zooxanthellae are unable to protect themselves by elevating antioxidant enzyme activity, a similar conclusion can be reached for cold bleaching. Alternatively, if cold stress bleaching is not associated with increased ROS production, then the bleaching may simply be the result of the expulsion of zooxanthellae that have lost photosynthetic function at low temperature. However, additional experiments should test these enzymes at other time points, either earlier in the bleaching process or with other antioxidants (glutathione peroxidase, ascorbate peroxidase, etc.), before one can conclude that there is no antioxidant response in cold stress.

We observed decreases in zooxanthella density, gross production, and calcification, pointing to a common physiological response to bleaching under hot and cold stresses. Decreased calcification is consistent with the results of previous cold stress experiments^15^. Several studies have reported that photosynthesis and calcification rates are significantly correlated^25^,^26^. The declines in photochemical efficiency and zooxanthella density may have contributed to the large reductions in coral growth, as the lowest rates of calcification at 13 °C corresponded to low photosynthetic activity. Values of respiratory ETSA suggested that potential respiration of host coral decreases under cold stress. We performed all ETSA measurements at 25 °C to compare the state of the enzymatic system under analogous conditions; the actual ETSA at 13 °C would have been even lower, due to its positive correlation with temperature^27^. The decrease in potential respiration rates, as measured by ETSA, and in actual respiration rates could be due to a continuous decrease in the amount of substrate available for respiration, potentially contributing to the decreased calcification rates by limiting the energy available for the calcification process. The decrease in substrate available for host respiration can be attributed to a reduction in the amount of translocated photosynthate or a decrease in the host reserve. Gradual decreases in lipid content have been observed under low temperature stress in tropical corals and at low light levels for temperate corals^28^, suggesting that lipid reserves play an important role in the mechanisms of, and resilience to, cold bleaching. Corals may also gradually use their lipid reserve to survive with minimum levels of metabolism and respiration rates through the winter, meeting their energy requirements through heterotrophy, which might also be an alternative sporadic source during winter^29^. The differences in heterotrophic capacity among coral species might also determine differences in cold stress resilience. Finally, cold stress bleaching could serve as a strategy to reduce the energy burden on the host due to energy consumption by zooxanthellae^30^,^31^, which have low photosynthetic efficiency at low temperatures^11^,^13^. Therefore, cold bleaching might play a crucial role in the survival of *A*. *pruinosa* at the low temperatures experienced during winter. The expulsion of zooxanthellae removes the energy burden from the host. As a result, the coral can survive the cold winter by maintaining minimal metabolism. While the loss of zooxanthellae can be seen as a way the coral protects itself, if stressful temperatures last for too long or are too severe, bleaching will result in coral death.

Photodamage at low temperatures interferes with the normal replacement rate of D1 protein in the turnover-repair cycle^32^. The observed recovery of the photosynthetic efficiency of *A*. *pruinosa* when the seawater temperature was restored suggests that the turnover-repair cycle is reactivated with increased temperature. Moreover, we observed an increase in the number of zooxanthellae during the recovery period, showing that the mutualistic relationship between coral and zooxanthellae was fully reinstituted at temperatures >20 °C.

Cold events can occur abruptly in both temperate and tropical zones, causing coral bleaching and high mortality. Our results demonstrated that corals are very sensitive to cold stress due to a lack of protection against cold stress-induced damage. In addition, differences in the resilience among different acroporid species can help to explain their geographic distribution. Cold bleaching of corals could represent a strategy in some species to survive the winter, as bleaching removes the energetic burden of zooxanthellae under low temperatures. Nevertheless, due to the high mortality rates of two of the three tested species, cold temperature events could severely affect the poleward range expansion of corals^1^,^33^.

## Methods

### Coral specimens

One colony of each coral species: *Acropora pruinosa*, *A*. *hyacinthus*, and *A*. *solitaryensis* were collected from a coastal region of Shirahama (Supplementary Fig. S1) in Wakayama Prefecture, Japan (with permission from Wakayama Prefecture). The *Symbiodinium* spp. in these *Acropora* all dominated by clade C, as confirmed using restriction fragment length polymorphism (RFLP) alanysis^34^. Coral fragment tips (ca. 3 cm long) from each colony were cut and attached to an acrylic plate. Fragments were kept at 18 °C for 2 weeks in an aquarium with running seawater to allow for recovery from sampling and cutting stress.

### Experimental design

A total of 27 fragments (9 fragments with same genotype per species, n = 3 per treatment) were selected for the experiment and distributed randomly among three 20 L thermostatic aquariums, initial temperature 18 °C, with continuously supply of seawater. Turn-over of the water in the aquariums was 3 hours (Supplementary Fig. S2). This experimental setup may not follow the wide variety of physiological response of corals with deferent genotypes to temperature stress. Light was provided by metal halide lamps (70 W, Kamihata) with a constant photon flux density (100 or 0 μmol m^−2^ s^−1^ during a 12:12-h light:dark cycle, measured with a LI-COR 2PI photometer). Water temperatures of two of the aquariums were gradually (1 °C per day) changed to the target temperature of 13 °C or 23 °C from 18 °C over 5 days, and one aquarium temperature was maintained at 18 °C. After the treatment temperatures were reached, 10-day incubations were conducted for each treatment. At the end of the incubation, metabolic and physiological characteristics of surviving fragments were measured in independent chambers. Photography and pulse amplitude modulated (PAM) fluorometric measurements were performed on days 0, 5, and 10 of the incubation and the fragments were shuffled within treatments at that time.

### Measurements of metabolic and physiological characteristics

After a minimum of 30 min of dark incubation, the maximum quantum efficiency of Photosystem II (*F*_*v*_*/F*_*m*_) was measured using a junior PAM (Walz, Germany). The settings for junior PAM were as follow: measuring light 8, saturation light 8 and gain 1. Photographs of the fragments were taken after PAM measurements.

For measurements of the metabolic processes of corals, the water supply to coral fragments was stopped for 3 h under light conditions and for three additional hours under dark conditions. During these periods, water movement was provided by magnetic stirrers. Total alkalinity (TA) and pH of the collected seawater were determined using the Gran plot method with a TA titrator (ATT-05, Kimoto) and a pH meter (Orion 4 stars, Thermo Scientific), respectively. The rates of gross production, respiration, and calcification were calculated by changes in pH and TA while the water exchange was stopped^35^.

Coral tissues were removed from the skeleton using an air jet filled with ice-cold 100 mmol l^−1^ phosphate buffer containing 10 g l^−1^ of NaCl and were then homogenized on ice. Before stripping the tissue, all samples were washed with 0.7 M NaCl solution to remove loosely attached plankton and other organisms. A small portion of the homogenate was used to count zooxanthellae density using a Neubauer hemocytometer. The non-fixed samples were then separated into host coral tissue and endosymbiont zooxanthellae (zoox) by centrifugation at 600 × g for 10 min. The supernatant was used to analyze the respiratory electron transport system activity (ETSA) and protein and enzyme activities of the host coral. After washing the zooxanthellae pellet with NaCl solution by two successive centrifugation–re-suspension cycles, zooxanthellae were lysed by sonication in phosphate buffer with 0.025% Triton-X100 for 10 min in an ice bath. After sonication, the suspension was centrifuged at 14,000 × g for 10 min and used as the zooxanthella fraction for protein and enzyme assays. The ETSA assay^27^ of host extract was conducted after 5 min of sonication (VP-050, TAITEC) at 25% power on ice. The SOD activities of host and zoox fractions were assayed spectrophotometrically^36^,^37^. Standards for activity were prepared using bovine erythrocytic SOD (Sigma) for each set of samples. The CAT activity of host extract was measured by the depletion of H_2_O_2_ at 240 nm^38^. All assays were conducted at 25 °C immediately after sampling, and enzyme activity was expressed as units (U) per mg protein. Protein content was determined using the Bradford assay^39^.

### Recovery from cold stress

We investigated the recovery of one specimen *A*. *pruinosa* from cold bleaching provoked with incubation for 10 days at 13 °C. After the cold stress period, the water temperature was increased by 2 °C per 10 days from 13 °C to 23 °C, and corals were maintained for 50 days at 23 °C. Water temperatures during the recovery period were recorded using Hobo pendant data loggers (Onset, USA). The state of the corals during recovery was evaluated by their coloration and maximum photochemical yield (*F*_*v*_*/F*_*m*_).

### Statistical analysis

All experiments, including recovery testing, were conducted with three replicates per treatment. All data were normalized to the surface areas of the fragments or by protein content. *Post hoc* differences were assessed using Tukey-Kramer honestly significant difference (HSD) tests (JMP 8.0, SAS). To determine the recovery state, Dunnett’s test was conducted by comparison with initial values as a control.

## Additional Information

**How to cite this article**: Higuchi, T. *et al.* The northern limit of corals of the genus *Acropora* in temperate zones is determined by their resilience to cold bleaching. *Sci. Rep.* **5**, 18467; doi: 10.1038/srep18467 (2015).

## Supplementary Material

Supplementary Information

## Figures and Tables

**Table 1:** Zooxanthellae density, coral metabolism (gross production, respiration, calcification), respiratory electron transport system activity (ETSA), and antioxidant enzyme activities (superoxide dismutase: SOD, catalase: CAT) of acroporid corals after 10 days at 23, 18, and 13 °C (means ± SE, n = 3 per treatment).

	*A. pruinosa*			*A. hyacinthus*		*A. solitaryensis*	
	23 °C	18 °C	13 °C	23 °C	18 °C	23 °C	18 °C
Zoox density (× 10^5^ cells cm^−2^)	9.8 ± 3.6	8.0 ± 0.5	2.2 ± 0.7*	5.5 ± 1.5	3.5 ± 0.6	8.0 ± 1.2	9.9 ± 1.3
Gross production (μmol cm^−2^ h^−1^)	8.3 ± 0.8	11.9 ± 0.8	3.0 ± 1.0*	6.2 ± 1.2	8.9 ± 0.9	7.2 ± 1.1	6.5 ± 2.0
Respiration (μmol cm^−2^ h^−1^)	6.1 ± 0.7	7.3 ± 0.9	0.5 ± 0.3*	5.5 ± 0.9	7.3 ± 0.8	6.0 ± 1.1	4.8 ± 1.6
Calcification (μmol cm^−2^ h^−1^)	2.7 ± 0.3	3.4 ± 0.6	0.3 ± 0.3*	2.2 ± 0.7	1.5 ± 0.1	1.6 ± 0.4	1.1 ± 0.6
ETSA (μmol O_2_ h^−1^ mg protein^−1^)	0.52 ± 0.02	0.58 ± 0.04	0.39 ± 0.03*	0.46 ± 0.07	0.53 ± 0.09	0.40 ± 0.05	0.31 ± 0.02
SOD host (Unit mg protein^−1^)	2.99 ± 0.05	3.07 ± 0.23	1.76 ± 0.08*	4.56 ± 0.06	4.86 ± 0.19	6.03 ± 0.09 *	4.45 ± 0.20
SOD zoox (Unit mg protein^−1^)	2.15 ± 0.17	1.64 ± 0.07	1.62 ± 0.14	4.89 ± 0.15*	4.18 ± 0.21	4.89 ± 0.33*	3.14 ± 0.06
CAT host (Unit mg protein^−1^)	29.5 ± 4.2	29.4 ± 2.9	19.8 ± 1.5*	17.2 ± 3.4	20.1 ± 1.8	11.9 ± 0.9	12.3 ± 0.9

Asterisks (*) in each column indicate significant differences compared to control treatments (18 °C) on the same specimen (Tukey-Kramer HSD, P < 0.05).
