# Supplementary material for: The northern limit of corals of the genus Acropora in temperate zones is determined by their resilience to cold bleaching
Source: Sci Rep. 2015 Dec 18;5:18467. doi: 10.1038/srep18467 (PMC4683436; doi:10.1038/srep18467)
Supplement: Supplementary Information [file srep18467-s1.pdf]

[Supplementary Information]

The northern limit of corals of the genus *Acropora* in temperate zones is determined by their resilience to cold bleaching

Tomihiko Higuchi, Sylvain Agostini, Beatriz Estela Casareto, Yoshimi Suzuki & Ikuko Yuyama

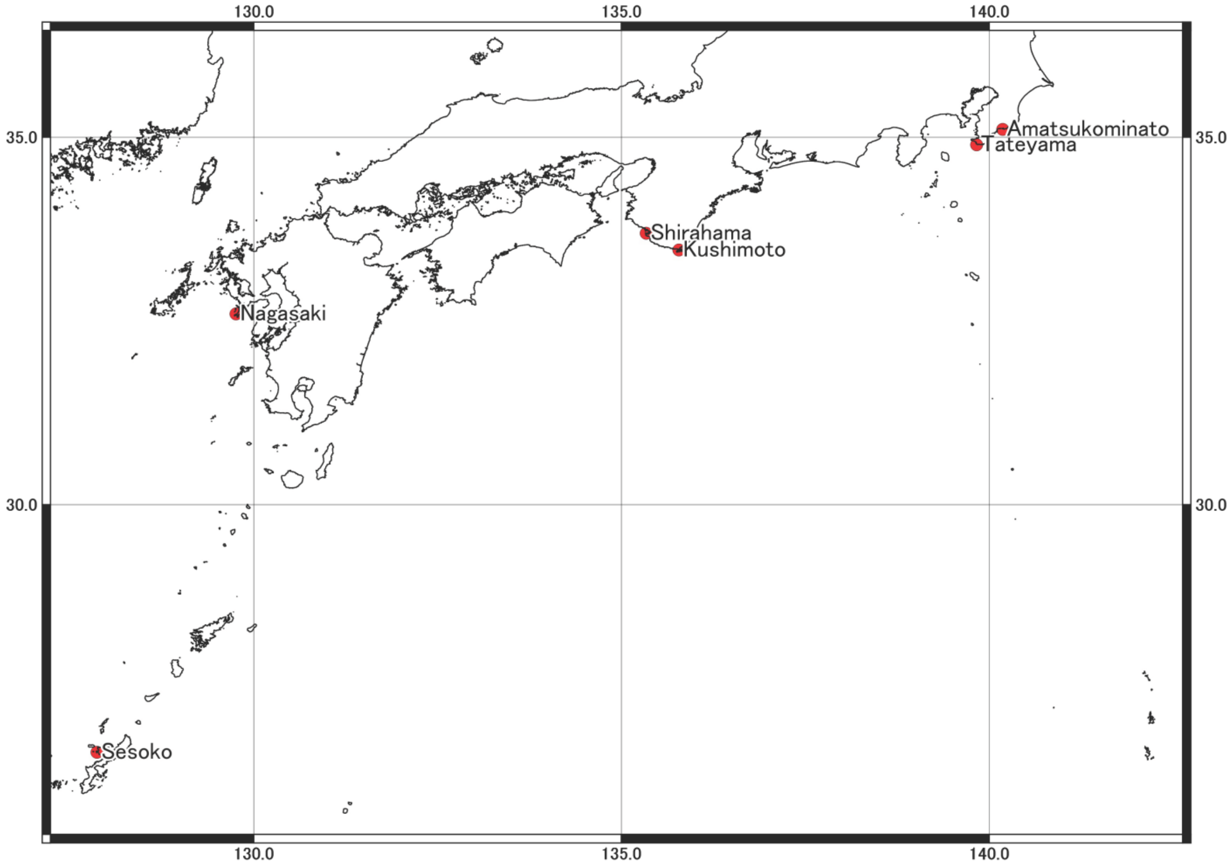

Supplementary Figure S1

A map of Japan with locations where described in main text (X: east longitude, Y: north latitude). The map was created using the free software Quantum Geographic Information System (QGIS2.10, Quantum GIS Development Team), the World Vector Shoreline dataset[40,41] released under the GNU Lesser General Public License v3 ("GSHHG - A Global Self-consistent, Hierarchical, High-resolution Geography Database)[42] and publicly available latitudes and longitudes of the different locations.

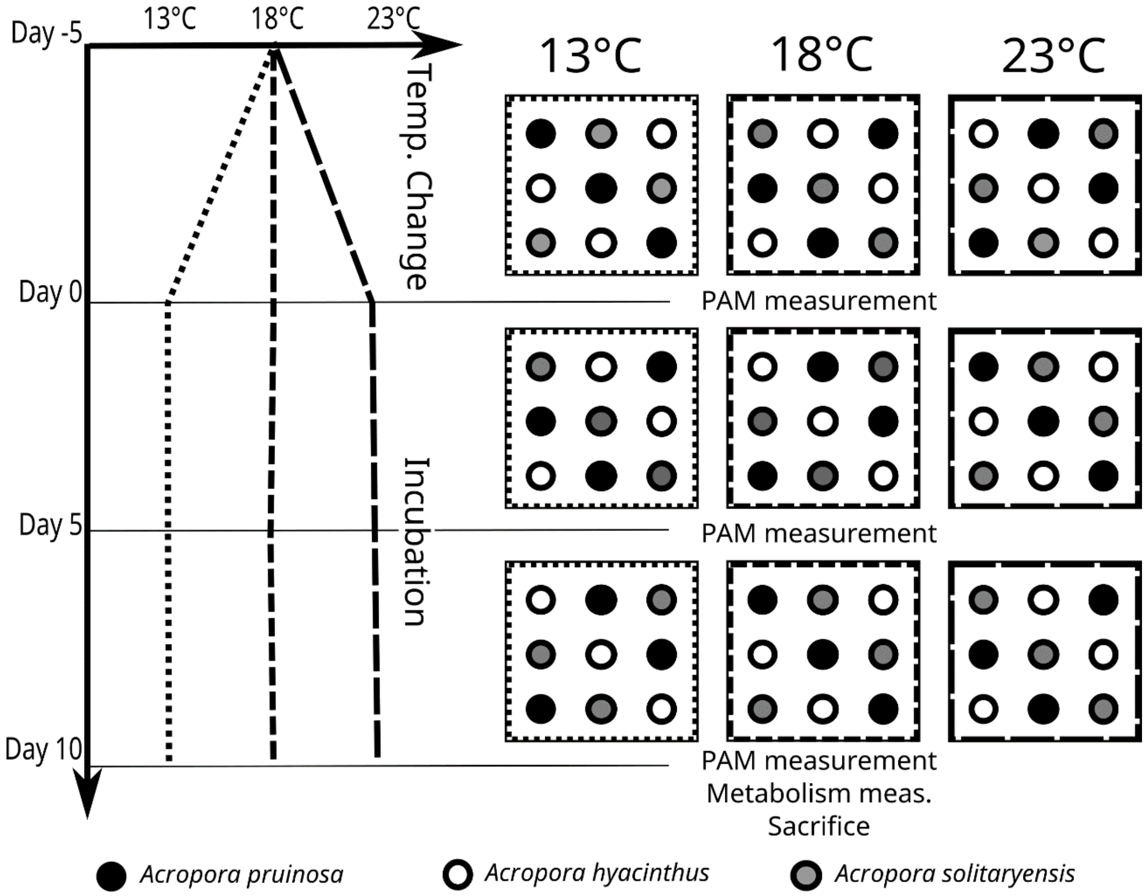

Supplementary Figure S2

Schematic diagram of experimental design. A total of 27 branches, n = 3 per species and per temperature, were selected for the experiment and distributed randomly among three 20 L thermostatic aquariums, initial temperature 18°C, with continuously supply of seawater. Turn-over of the water in the aquariums was 3 hours.

Supplementary Table S1

Monthly averages of sea surface temperature around Japan in 2013. Temperature at Sesoko (26°N) was provided from "RECORD OF COASTAL OBSERVATION AT THE SESOKO STATION, TROPICAL BIOSPHERE RESEARCH CENTER, THE UNIVERSITY OF THE RYUKYUS", at Shirahama(33°N) was reported by Shirahama aquarium, Kyoto University[8], at Tateyama (34°N) was reported at database of Kanagawa Prefectural Fisheries Technology Center.

(^{o}C)

	Jan	Feb	Mar	Apr	May	Jun	Jul	Aug	Sep	Oct	Nov	Dec
Sesoko	20.2	20.5	21.0	21.2	23.3	26.7	28.8	29.3	27.8	25.6	23.2	20.7
Shirahama	14.7	14.4	16.4	18.0	20.4	23.6	26.6	28.0	26.1	24.2	19.6	15.6
Tateyama	13.3	13.6	15.2	17.4	18.5	20.4	23.2	25.6	25.1	22.4	18.2	15.9

Supplementary References

40. Wessel, P. & Smith, W.H.F. A global, self-consistent, hierarchical, high-resolution shoreline database. *J. Geophys. Res.* **101**, 8741-8743 (1996).

41. NOAA National Geophysical Data Center, Coastline extracted GSHHG, Available at http://www.ngdc.noaa.gov/mgg/shorelines/gshhs.html, (Accessed 14th September 2015).

42. GSHHG - A Global Self-consistent, Hierarchical, High-resolution Geography Database. Available at http://www.soest.hawaii.edu/pwessel/gshhg/. (Accessed 14th September 2015).
